# Aqua­bis(2,2′-bipyridine-κ^2^ *N*,*N*′)(1*H*-indole-2-carboxyl­ato-κ*O*)nickel(II) 1*H*-indole-2-carboxyl­ate dihydrate

**DOI:** 10.1107/S1600536808041391

**Published:** 2008-12-13

**Authors:** Bi-Song Zhang, Zhen-Xiang Liu, Li-Hua Liu, Tao Pan, Su-Fang Ye

**Affiliations:** a College of Materials Science and Chemical Engineering, Jinhua College of Profession and Technology, Jinhua, Zhejiang 321017, People’s Republic of China

## Abstract

The hydro­thermal reaction of Ni_2_(OH)_2_CO_3_ with 2,2′-bipyridine and 2-indolyl-formic acid in CH_3_OH/H_2_O at 423 K for 7 d produced the novel Ni^II^ complex [Ni(C_9_H_6_NO_2_)(C_10_H_8_N_2_)_2_(H_2_O)](C_9_H_6_NO_2_)·2H_2_O. The asymmetric unit of the title compound consists of a monovalent [Ni(*L*)(bpy)_2_(H_2_O)]^+^ cation (bpy is 2,2′-bipyridine and *L* is 1*H*-indole-2-carboxyl­ate), an *L* anion and two solvent water mol­ecules. In the [Ni(*L*)(bpy)_2_(H_2_O)]^+^ cations, the Ni atom coordinates to four N atoms from the two bpy ligands and two O atoms, one from a *L* anion and the other from a water mol­ecule to complete an significantly distorted NiN_4_O_2_ octa­hedron. The coordinated and solvate water mol­ecules form an extensive series of O—H⋯O hydrogen bonds. N—H⋯O and C—H⋯O hydrogen bonds are also present and the mol­ecules are inter­linked, forming a three-dimensional network.

## Related literature

For other complexes of the 1*H*-indole-2-carboxyl­ate ligand, see: Lou & Zhang (2007 ▶); Zhang & Ying (2005 ▶). For related structures, see: Zhang (2004 ▶, 2005 ▶, 2006*a* ▶,*b* ▶,*c* ▶); Zhang *et al.* (2005 ▶).
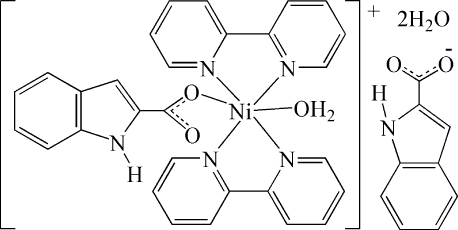


## Experimental

### 

#### Crystal data

[Ni(C_9_H_6_NO_2_)(C_10_H_8_N_2_)_2_(H_2_O)](C_9_H_6_NO_2_)·2H_2_O
*M* *_r_* = 745.42
Triclinic, 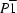

*a* = 12.499 (8) Å
*b* = 13.128 (9) Å
*c* = 13.477 (9) Å
α = 95.389 (9)°
β = 114.166 (9)°
γ = 117.804 (8)°
*V* = 1669.7 (19) Å^3^
*Z* = 2
Mo *K*α radiation
μ = 0.64 mm^−1^
*T* = 293 (2) K
0.40 × 0.21 × 0.13 mm

#### Data collection

Bruker SMART CCD area-detector diffractometer
Absorption correction: multi-scan (*SADABS*; Sheldrick, 1996 ▶) *T*_min_ = 0.848, *T*_max_ = 0.920
9411 measured reflections
6750 independent reflections
5675 reflections with *I* > 2σ(*I*)
*R*_int_ = 0.026

#### Refinement

*R*[*F* ^2^ > 2σ(*F* ^2^)] = 0.050
*wR*(*F* ^2^) = 0.148
*S* = 1.07
6750 reflections
470 parameters
H-atom parameters constrained
Δρ_max_ = 1.23 e Å^−3^
Δρ_min_ = −0.88 e Å^−3^

### 

Data collection: *SMART* (Bruker, 1998 ▶); cell refinement: *SAINT* (Bruker, 1998 ▶); data reduction: *SAINT*; program(s) used to solve structure: *SHELXS97* (Sheldrick, 2008 ▶); program(s) used to refine structure: *SHELXL97* (Sheldrick, 2008 ▶); molecular graphics: *SHELXTL* (Sheldrick, 2008 ▶); software used to prepare material for publication: *SHELXTL*.

## Supplementary Material

Crystal structure: contains datablocks I, global. DOI: 10.1107/S1600536808041391/sj2561sup1.cif

Structure factors: contains datablocks I. DOI: 10.1107/S1600536808041391/sj2561Isup2.hkl

Additional supplementary materials:  crystallographic information; 3D view; checkCIF report

## Figures and Tables

**Table 1:** Hydrogen-bond geometry (Å, °)

*D*—H⋯*A*	*D*—H	H⋯*A*	*D*⋯*A*	*D*—H⋯*A*
O1—H1*A*⋯O4	0.82	1.89	2.647 (3)	154.0
O2—H2*A*⋯O5	0.82	1.91	2.732 (4)	178.3
O2—H2*B*⋯O4	0.82	2.10	2.885 (4)	161.7
O7—H7*A*⋯O5	0.81	2.42	2.994 (4)	129.0
N6—H*N*6⋯O6^i^	0.86	1.99	2.814 (3)	159.0
O1—H1*B*⋯O6^ii^	0.82	1.93	2.750 (3)	174.2
O1—H1*B*⋯O5^ii^	0.82	2.60	3.166 (3)	127.6
C4—H4⋯O2^iii^	0.93	2.54	3.400 (5)	155
C14—H14⋯O7^iv^	0.93	2.41	3.320 (4)	167

Symmetry codes: (i) 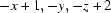; (ii) 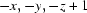; (iii) 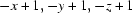; (iv) 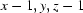.

**Fig. 1.**
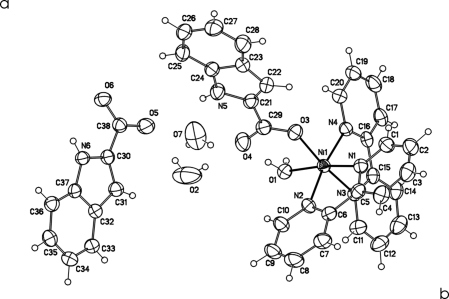
The asymmetric unit of (I), showing the atom-labelling scheme. Displacement ellipsoids are drawn at the 50% probability level.

**Fig. 2.**
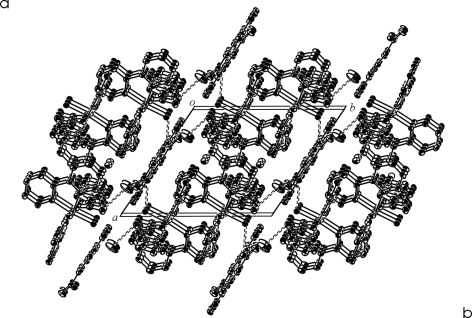
A packing diagram of the title complex, viewed along the c axis (the H atoms have been omitted). Hydrogen bonds are drawn as dashed lines.

## supplementary crystallographic information

### Comment

We have prepared the title complex, [Ni(H_2_O)(bpy)_2_(*L*)].*L*.2H_2_O [bpy = 2,2'-bipyridine, HL = 2-indolyl-formic acid] (I), and report its crystal structure here, Fig. 1. The title compound has a structure similar to those of complexes of halobenzoate ligands, *X*—C_6_H_4_COO^-^, where *X* is F,Cl,Br and I, (Zhang, 2004, 2005, 2006a,b,c; Zhang *et al.,* 2005).

The asymmetric unit of the title compound consists of a [Ni(H_2_O)(bpy)_2_(*L*)]^+^ cation, a 1H-indole-2-carboxylate anion and two solvent water molecules (Fig.1). In the cation, the Ni(1) atom is coordinated by four N atoms from two 2,2'-bipyridine ligands and two O atoms, one from a 1H-indole-2-carboxylate anion and the other from a water molecule to complete a significantly distorted NiN_4_O_2_ octahedron. The Ni—N bond lengths are in the range 2.066 (2) to 2.097 (2) Å, with  Ni—O distances 2.075 (2)Å and 2.080 (2)Å, Table 1.

The coordinated and solvate water molecules show extensive hydrogen bonding to the carboxylate O atoms of 2-indolyl-formic acid anions, Table 2. An N6—HN6···O6 hydrogen bond also forms.  In addition, weak C—H···O hydrogen bonds form between the O atoms of solvate water molecules and the H atoms of the 2,2'-bipyridine ligands. A combination of these strong and weak hydrogen bonding interactions link the molecules into a three-dimensional network, Fig 2.

### Experimental

Ni_2_(OH)_2_CO_3_ (0.12 g 0.57 mmol), 2,2'-bpy (0.04 g 0.26 mmol),2-indolyl- formic acid (0.06 g 0.37 mmol),15 ml CH_3_OH/H_2_O (1:2, *v*/*v*) were mixed and stirred for *ca*3.5 h., the resulting suspension was heated in a 23 ml Teflon-lined stainless steel autoclave at 423 K for 7 days. After the autoclave cooled to room temperature, the solid was filtered off. The resulting pink filtrate was allowed to stand at room temperature and slow evaporation over two weeks gave brown block-like crystals suitable for X-ray analysis.

### Refinement

C-bound H atoms were placed in calculated positions, with C—H = 0.93Å and *U*_iso_(H) = 1.2*U*_eq_(C),and were refined using the riding-model approximation. The H atoms of the water molecule were located in a difference Fourier map and refined with O—H distance restraints of 0.82 (1) Å and *U*_iso_(H) = 1.5*U*_eq_(O). The highest residual electron density was 1.04Å and the deepest hole 0.89Å from atom Ni1.

### Crystal data

**Table:**

[Ni(C_9_H_6_NO_2_)(C_10_H_8_N_2_)_2_(H_2_O)](C_9_H_6_NO_2_)·2H_2_O	*Z* = 2
*M**_r_* = 745.42	*F*(000) = 776
Triclinic, *P*1	*D*_x_ = 1.483 Mg m^−^^3^
Hall symbol: -P 1	Mo *K*α radiation, λ = 0.71073 Å
*a* = 12.499 (8) Å	Cell parameters from 238 reflections
*b* = 13.128 (9) Å	θ = 1.9–26.0°
*c* = 13.477 (9) Å	µ = 0.64 mm^−^^1^
α = 95.389 (9)°	*T* = 293 K
β = 114.166 (9)°	Block, brown
γ = 117.804 (8)°	0.40 × 0.21 × 0.13 mm
*V* = 1669.7 (19) Å^3^	

### Data collection

**Table:**

Bruker SMART CCD area-detector diffractometer	6750 independent reflections
Radiation source: fine-focus sealed tube	5675 reflections with *I* > 2σ(*I*)
graphite	*R*_int_ = 0.026
φ and ω scans	θ_max_ = 27.8°, θ_min_ = 1.9°
Absorption correction: multi-scan (*SADABS*; Sheldrick, 1996)	*h* = −15→15
*T*_min_ = 0.848, *T*_max_ = 0.920	*k* = −14→16
9411 measured reflections	*l* = −16→16

### Refinement

**Table:**

Refinement on *F*^2^	Secondary atom site location: difference Fourier map
Least-squares matrix: full	Hydrogen site location: inferred from neighbouring sites
*R*[*F*^2^ > 2σ(*F*^2^)] = 0.050	H-atom parameters constrained
*wR*(*F*^2^) = 0.148	*w* = 1/[σ^2^(*F*_o_^2^) + (0.1085*P*)^2^] where *P* = (*F*_o_^2^ + 2*F*_c_^2^)/3
*S* = 1.07	(Δ/σ)_max_ = 0.005
6750 reflections	Δρ_max_ = 1.23 e Å^−^^3^
470 parameters	Δρ_min_ = −0.88 e Å^−^^3^
0 restraints	Extinction correction: *SHELXL*, Fc^*^=kFc[1+0.001xFc^2^λ^3^/sin(2θ)]^-1/4^
Primary atom site location: structure-invariant direct methods	Extinction coefficient: 0.0027 (13)

### Special details

**Table:**

Geometry. All esds (except the esd in the dihedral angle between two l.s. planes) are estimated using the full covariance matrix. The cell esds are taken into account individually in the estimation of esds in distances, angles and torsion angles; correlations between esds in cell parameters are only used when they are defined by crystal symmetry. An approximate (isotropic) treatment of cell esds is used for estimating esds involving l.s. planes.
Refinement. Refinement of F^2^ against ALL reflections. The weighted R-factor wR and goodness of fit S are based on F^2^, conventional R-factors R are based on F, with F set to zero for negative F^2^. The threshold expression of F^2^ > 2sigma(F^2^) is used only for calculating R-factors(gt) etc. and is not relevant to the choice of reflections for refinement. R-factors based on F^2^ are statistically about twice as large as those based on F, and R- factors based on ALL data will be even larger.

### Fractional atomic coordinates and isotropic or equivalent isotropic displacement parameters (Å^2^)

**Table:**

	*x*	*y*	*z*	*U*_iso_*/*U*_eq_	
Ni1	0.04771 (3)	0.33215 (2)	0.29047 (2)	0.02858 (13)	
N1	0.1702 (2)	0.52501 (17)	0.35544 (16)	0.0333 (4)	
N2	0.2419 (2)	0.37219 (18)	0.31901 (17)	0.0336 (4)	
N3	−0.0299 (2)	0.33007 (17)	0.11931 (16)	0.0322 (4)	
N4	−0.1395 (2)	0.31369 (17)	0.25242 (16)	0.0321 (4)	
N5	0.1344 (2)	0.18354 (19)	0.67095 (17)	0.0420 (5)	
HN5	0.1015	0.1087	0.6350	0.050*	
N6	0.5831 (2)	0.01927 (17)	0.89144 (16)	0.0326 (4)	
HN6	0.5880	0.0182	0.9568	0.039*	
O1	−0.04967 (16)	0.14307 (14)	0.22692 (13)	0.0349 (4)	
H1A	−0.0315	0.1199	0.2825	0.052*	
H1B	−0.1357	0.1010	0.1955	0.052*	
O2	0.2771 (4)	0.0847 (4)	0.5299 (3)	0.1255 (14)	
H2A	0.2798	0.0714	0.5889	0.188*	
H2B	0.2340	0.1178	0.5126	0.188*	
O3	0.09785 (19)	0.32891 (16)	0.45695 (14)	0.0403 (4)	
O4	0.0692 (2)	0.14428 (18)	0.44129 (15)	0.0512 (5)	
O5	0.2852 (2)	0.0358 (2)	0.72467 (18)	0.0577 (5)	
O6	0.33868 (18)	−0.01581 (16)	0.88179 (15)	0.0408 (4)	
O7	0.4874 (3)	0.3091 (2)	0.8106 (3)	0.0908 (9)	
H7B	0.5398	0.3770	0.8090	0.136*	
H7A	0.4439	0.2478	0.7542	0.136*	
C1	0.1267 (3)	0.5974 (2)	0.3726 (2)	0.0416 (6)	
H1	0.0343	0.5605	0.3561	0.050*	
C2	0.2134 (3)	0.7250 (2)	0.4139 (2)	0.0479 (6)	
H2	0.1804	0.7729	0.4255	0.058*	
C3	0.3493 (3)	0.7785 (2)	0.4372 (2)	0.0497 (7)	
H3	0.4098	0.8638	0.4652	0.060*	
C4	0.3965 (3)	0.7059 (2)	0.4189 (2)	0.0456 (6)	
H4	0.4883	0.7415	0.4343	0.055*	
C5	0.3036 (2)	0.5785 (2)	0.37713 (19)	0.0350 (5)	
C6	0.3433 (2)	0.4923 (2)	0.35529 (19)	0.0338 (5)	
C7	0.4745 (3)	0.5295 (3)	0.3708 (2)	0.0458 (6)	
H7	0.5428	0.6126	0.3938	0.055*	
C8	0.5028 (3)	0.4418 (3)	0.3517 (3)	0.0520 (7)	
H8	0.5907	0.4652	0.3626	0.062*	
C9	0.3989 (3)	0.3189 (3)	0.3161 (2)	0.0476 (6)	
H9	0.4156	0.2583	0.3032	0.057*	
C10	0.2701 (3)	0.2880 (2)	0.3001 (2)	0.0415 (6)	
H10	0.1997	0.2053	0.2752	0.050*	
C11	0.0317 (3)	0.3393 (2)	0.0559 (2)	0.0423 (6)	
H11	0.1170	0.3456	0.0888	0.051*	
C12	−0.0259 (3)	0.3397 (3)	−0.0549 (2)	0.0495 (7)	
H12	0.0193	0.3458	−0.0965	0.059*	
C13	−0.1513 (3)	0.3311 (2)	−0.1035 (2)	0.0506 (7)	
H13	−0.1922	0.3313	−0.1787	0.061*	
C14	−0.2165 (3)	0.3221 (2)	−0.0404 (2)	0.0424 (6)	
H14	−0.3005	0.3181	−0.0716	0.051*	
C15	−0.1548 (2)	0.31915 (19)	0.07050 (19)	0.0328 (5)	
C16	−0.2200 (2)	0.30411 (18)	0.14345 (19)	0.0313 (5)	
C17	−0.3542 (2)	0.2789 (2)	0.1037 (2)	0.0399 (5)	
H17	−0.4096	0.2697	0.0275	0.048*	
C18	−0.4041 (3)	0.2679 (2)	0.1790 (3)	0.0471 (6)	
H18	−0.4937	0.2513	0.1542	0.057*	
C19	−0.3208 (3)	0.2816 (2)	0.2905 (3)	0.0443 (6)	
H19	−0.3518	0.2767	0.3429	0.053*	
C20	−0.1910 (3)	0.3025 (2)	0.3239 (2)	0.0383 (5)	
H20	−0.1361	0.3094	0.3992	0.046*	
C21	0.1409 (3)	0.2719 (2)	0.6209 (2)	0.0379 (5)	
C22	0.1981 (3)	0.3790 (2)	0.7048 (2)	0.0408 (6)	
H22	0.2128	0.4521	0.6936	0.049*	
C23	0.2310 (3)	0.3590 (2)	0.8116 (2)	0.0370 (5)	
C24	0.1892 (3)	0.2352 (2)	0.7879 (2)	0.0366 (5)	
C25	0.2055 (3)	0.1829 (2)	0.8741 (2)	0.0444 (6)	
H25	0.1765	0.1008	0.8569	0.053*	
C26	0.2667 (3)	0.2583 (2)	0.9862 (2)	0.0446 (6)	
H26	0.2780	0.2260	1.0455	0.054*	
C27	0.3118 (3)	0.3818 (3)	1.0123 (2)	0.0488 (7)	
H27	0.3535	0.4302	1.0888	0.059*	
C28	0.2961 (3)	0.4334 (2)	0.9277 (2)	0.0502 (7)	
H28	0.3279	0.5161	0.9466	0.060*	
C29	0.0993 (2)	0.2451 (2)	0.4966 (2)	0.0387 (5)	
C30	0.4817 (2)	0.0220 (2)	0.8002 (2)	0.0342 (5)	
C31	0.5085 (3)	0.0230 (2)	0.7109 (2)	0.0374 (5)	
H31	0.4562	0.0253	0.6394	0.045*	
C32	0.6304 (2)	0.0198 (2)	0.7472 (2)	0.0336 (5)	
C33	0.7085 (3)	0.0185 (2)	0.6963 (2)	0.0384 (5)	
H33	0.6791	0.0158	0.6200	0.046*	
C34	0.8288 (3)	0.0215 (2)	0.7610 (2)	0.0437 (6)	
H34	0.8824	0.0234	0.7286	0.052*	
C35	0.8722 (3)	0.0216 (2)	0.8743 (2)	0.0443 (6)	
H35	0.9535	0.0227	0.9155	0.053*	
C36	0.7973 (3)	0.0202 (2)	0.9269 (2)	0.0381 (5)	
H36	0.8264	0.0203	1.0024	0.046*	
C37	0.6758 (2)	0.01867 (19)	0.86157 (19)	0.0328 (5)	
C38	0.3603 (2)	0.0142 (2)	0.8030 (2)	0.0356 (5)	

### Atomic displacement parameters (Å^2^)

**Table:**

	*U*^11^	*U*^22^	*U*^33^	*U*^12^	*U*^13^	*U*^23^
Ni1	0.02652 (19)	0.03213 (19)	0.02880 (18)	0.01791 (14)	0.01357 (14)	0.01124 (12)
N1	0.0342 (10)	0.0333 (10)	0.0309 (10)	0.0188 (8)	0.0158 (8)	0.0108 (7)
N2	0.0290 (10)	0.0372 (10)	0.0348 (10)	0.0210 (8)	0.0138 (8)	0.0114 (8)
N3	0.0321 (10)	0.0344 (10)	0.0294 (9)	0.0188 (8)	0.0150 (8)	0.0111 (7)
N4	0.0333 (10)	0.0339 (10)	0.0329 (10)	0.0210 (8)	0.0171 (8)	0.0126 (8)
N5	0.0545 (13)	0.0401 (11)	0.0338 (10)	0.0288 (10)	0.0214 (10)	0.0148 (8)
N6	0.0334 (10)	0.0351 (10)	0.0319 (10)	0.0193 (8)	0.0183 (8)	0.0126 (8)
O1	0.0324 (8)	0.0352 (8)	0.0332 (8)	0.0176 (7)	0.0150 (7)	0.0125 (7)
O2	0.133 (3)	0.273 (5)	0.095 (2)	0.164 (3)	0.087 (2)	0.116 (3)
O3	0.0456 (10)	0.0482 (10)	0.0321 (9)	0.0296 (8)	0.0186 (8)	0.0192 (7)
O4	0.0678 (13)	0.0569 (12)	0.0365 (9)	0.0437 (10)	0.0216 (9)	0.0195 (8)
O5	0.0598 (13)	0.0922 (15)	0.0621 (13)	0.0577 (12)	0.0414 (11)	0.0479 (12)
O6	0.0356 (9)	0.0513 (10)	0.0395 (9)	0.0242 (8)	0.0220 (8)	0.0185 (8)
O7	0.0528 (14)	0.0629 (15)	0.121 (2)	0.0271 (12)	0.0237 (15)	0.0274 (14)
C1	0.0440 (14)	0.0406 (13)	0.0456 (14)	0.0257 (11)	0.0250 (12)	0.0146 (11)
C2	0.0625 (18)	0.0416 (14)	0.0484 (15)	0.0330 (13)	0.0306 (14)	0.0159 (11)
C3	0.0553 (17)	0.0321 (13)	0.0458 (15)	0.0180 (12)	0.0213 (13)	0.0111 (11)
C4	0.0391 (14)	0.0378 (13)	0.0452 (14)	0.0158 (11)	0.0168 (11)	0.0137 (11)
C5	0.0334 (12)	0.0370 (12)	0.0298 (11)	0.0178 (10)	0.0141 (9)	0.0130 (9)
C6	0.0273 (11)	0.0415 (12)	0.0303 (11)	0.0186 (10)	0.0133 (9)	0.0140 (9)
C7	0.0333 (13)	0.0511 (15)	0.0488 (15)	0.0204 (11)	0.0215 (11)	0.0173 (12)
C8	0.0372 (14)	0.074 (2)	0.0561 (17)	0.0350 (14)	0.0279 (13)	0.0258 (14)
C9	0.0466 (15)	0.0642 (18)	0.0526 (16)	0.0412 (14)	0.0289 (13)	0.0236 (13)
C10	0.0399 (14)	0.0441 (14)	0.0482 (14)	0.0275 (11)	0.0241 (12)	0.0159 (11)
C11	0.0426 (14)	0.0480 (14)	0.0399 (13)	0.0246 (11)	0.0247 (11)	0.0161 (11)
C12	0.0605 (18)	0.0504 (15)	0.0391 (14)	0.0270 (14)	0.0310 (13)	0.0177 (11)
C13	0.0613 (18)	0.0483 (15)	0.0306 (13)	0.0262 (13)	0.0193 (12)	0.0146 (11)
C14	0.0433 (14)	0.0420 (13)	0.0349 (12)	0.0245 (11)	0.0134 (11)	0.0152 (10)
C15	0.0334 (12)	0.0252 (10)	0.0311 (11)	0.0152 (9)	0.0115 (9)	0.0076 (8)
C16	0.0297 (11)	0.0246 (10)	0.0341 (11)	0.0160 (9)	0.0116 (9)	0.0082 (8)
C17	0.0323 (12)	0.0362 (12)	0.0471 (14)	0.0219 (10)	0.0140 (10)	0.0135 (10)
C18	0.0362 (13)	0.0454 (14)	0.0689 (18)	0.0275 (12)	0.0284 (13)	0.0214 (13)
C19	0.0453 (15)	0.0446 (14)	0.0620 (17)	0.0295 (12)	0.0360 (13)	0.0241 (12)
C20	0.0424 (14)	0.0424 (13)	0.0427 (13)	0.0274 (11)	0.0264 (11)	0.0193 (10)
C21	0.0368 (13)	0.0466 (14)	0.0369 (12)	0.0256 (11)	0.0201 (10)	0.0202 (10)
C22	0.0475 (14)	0.0416 (13)	0.0451 (14)	0.0286 (12)	0.0271 (12)	0.0228 (11)
C23	0.0390 (13)	0.0388 (12)	0.0398 (13)	0.0245 (10)	0.0217 (11)	0.0160 (10)
C24	0.0401 (13)	0.0420 (13)	0.0315 (11)	0.0253 (11)	0.0181 (10)	0.0147 (9)
C25	0.0593 (17)	0.0445 (14)	0.0401 (13)	0.0344 (13)	0.0263 (12)	0.0211 (11)
C26	0.0509 (16)	0.0561 (16)	0.0374 (13)	0.0348 (13)	0.0236 (12)	0.0238 (11)
C27	0.0549 (17)	0.0533 (16)	0.0342 (13)	0.0288 (13)	0.0220 (12)	0.0121 (11)
C28	0.0653 (18)	0.0394 (14)	0.0460 (15)	0.0297 (13)	0.0285 (14)	0.0127 (11)
C29	0.0337 (12)	0.0518 (15)	0.0355 (12)	0.0274 (11)	0.0165 (10)	0.0195 (11)
C30	0.0369 (12)	0.0299 (11)	0.0367 (12)	0.0187 (9)	0.0196 (10)	0.0115 (9)
C31	0.0380 (13)	0.0433 (13)	0.0360 (12)	0.0250 (11)	0.0198 (10)	0.0158 (10)
C32	0.0363 (12)	0.0330 (11)	0.0352 (12)	0.0205 (10)	0.0196 (10)	0.0129 (9)
C33	0.0459 (14)	0.0433 (13)	0.0365 (12)	0.0285 (11)	0.0244 (11)	0.0175 (10)
C34	0.0456 (14)	0.0494 (15)	0.0524 (15)	0.0315 (12)	0.0315 (13)	0.0210 (12)
C35	0.0425 (14)	0.0537 (15)	0.0488 (15)	0.0346 (12)	0.0227 (12)	0.0237 (12)
C36	0.0375 (13)	0.0403 (13)	0.0374 (12)	0.0223 (11)	0.0188 (10)	0.0154 (10)
C37	0.0373 (12)	0.0283 (11)	0.0351 (11)	0.0184 (9)	0.0200 (10)	0.0122 (9)
C38	0.0335 (12)	0.0358 (12)	0.0386 (12)	0.0200 (10)	0.0184 (10)	0.0139 (9)

### Geometric parameters (Å, °)

**Table:**

Ni1—N4	2.064 (2)	C10—H10	0.9300
Ni1—O1	2.075 (2)	C11—C12	1.369 (4)
Ni1—O3	2.078 (2)	C11—H11	0.9300
Ni1—N2	2.079 (2)	C12—C13	1.371 (4)
Ni1—N1	2.095 (2)	C12—H12	0.9300
Ni1—N3	2.096 (2)	C13—C14	1.378 (4)
N1—C1	1.341 (3)	C13—H13	0.9300
N1—C5	1.352 (3)	C14—C15	1.387 (3)
N2—C10	1.339 (3)	C14—H14	0.9300
N2—C6	1.347 (3)	C15—C16	1.486 (3)
N3—C11	1.345 (3)	C16—C17	1.388 (3)
N3—C15	1.350 (3)	C17—C18	1.379 (4)
N4—C20	1.346 (3)	C17—H17	0.9300
N4—C16	1.350 (3)	C18—C19	1.367 (4)
N5—C24	1.377 (3)	C18—H18	0.9300
N5—C21	1.382 (3)	C19—C20	1.367 (4)
N5—HN5	0.8598	C19—H19	0.9300
N6—C30	1.376 (3)	C20—H20	0.9300
N6—C37	1.377 (3)	C21—C22	1.367 (4)
N6—HN6	0.8591	C21—C29	1.485 (3)
O1—H1A	0.8201	C22—C23	1.413 (3)
O1—H1B	0.8190	C22—H22	0.9300
O2—H2A	0.8192	C23—C24	1.410 (4)
O2—H2B	0.8176	C23—C28	1.412 (4)
O3—C29	1.272 (3)	C24—C25	1.396 (3)
O4—C29	1.253 (3)	C25—C26	1.384 (4)
O5—C38	1.253 (3)	C25—H25	0.9300
O6—C38	1.253 (3)	C26—C27	1.395 (4)
O7—H7B	0.8336	C26—H26	0.9300
O7—H7A	0.8125	C27—C28	1.372 (4)
C1—C2	1.389 (4)	C27—H27	0.9300
C1—H1	0.9300	C28—H28	0.9300
C2—C3	1.374 (4)	C30—C31	1.375 (3)
C2—H2	0.9300	C30—C38	1.489 (4)
C3—C4	1.385 (4)	C31—C32	1.420 (4)
C3—H3	0.9300	C31—H31	0.9300
C4—C5	1.392 (3)	C32—C33	1.409 (3)
C4—H4	0.9300	C32—C37	1.412 (3)
C5—C6	1.481 (3)	C33—C34	1.375 (4)
C6—C7	1.388 (4)	C33—H33	0.9300
C7—C8	1.385 (4)	C34—C35	1.395 (4)
C7—H7	0.9300	C34—H34	0.9300
C8—C9	1.382 (4)	C35—C36	1.381 (4)
C8—H8	0.9300	C35—H35	0.9300
C9—C10	1.376 (4)	C36—C37	1.398 (4)
C9—H9	0.9300	C36—H36	0.9300
			
N4—Ni1—O1	92.31 (7)	C14—C13—H13	120.2
N4—Ni1—O3	93.38 (7)	C13—C14—C15	118.9 (3)
O1—Ni1—O3	90.27 (7)	C13—C14—H14	120.5
N4—Ni1—N2	171.38 (7)	C15—C14—H14	120.5
O1—Ni1—N2	94.06 (7)	N3—C15—C14	121.5 (2)
O3—Ni1—N2	92.40 (7)	N3—C15—C16	115.51 (19)
N4—Ni1—N1	95.40 (8)	C14—C15—C16	123.0 (2)
O1—Ni1—N1	172.15 (7)	N4—C16—C17	121.6 (2)
O3—Ni1—N1	90.72 (7)	N4—C16—C15	114.8 (2)
N2—Ni1—N1	78.12 (8)	C17—C16—C15	123.6 (2)
N4—Ni1—N3	78.62 (7)	C18—C17—C16	119.0 (2)
O1—Ni1—N3	89.26 (7)	C18—C17—H17	120.5
O3—Ni1—N3	171.96 (7)	C16—C17—H17	120.5
N2—Ni1—N3	95.64 (8)	C19—C18—C17	119.4 (2)
N1—Ni1—N3	90.84 (7)	C19—C18—H18	120.3
C1—N1—C5	118.6 (2)	C17—C18—H18	120.3
C1—N1—Ni1	126.13 (17)	C20—C19—C18	119.1 (2)
C5—N1—Ni1	115.25 (16)	C20—C19—H19	120.4
C10—N2—C6	119.1 (2)	C18—C19—H19	120.4
C10—N2—Ni1	124.84 (16)	N4—C20—C19	122.8 (2)
C6—N2—Ni1	116.03 (16)	N4—C20—H20	118.6
C11—N3—C15	118.3 (2)	C19—C20—H20	118.6
C11—N3—Ni1	127.00 (18)	C22—C21—N5	108.9 (2)
C15—N3—Ni1	114.71 (14)	C22—C21—C29	130.2 (2)
C20—N4—C16	118.0 (2)	N5—C21—C29	120.7 (2)
C20—N4—Ni1	125.64 (16)	C21—C22—C23	108.0 (2)
C16—N4—Ni1	116.17 (15)	C21—C22—H22	126.0
C24—N5—C21	108.7 (2)	C23—C22—H22	126.0
C24—N5—HN5	125.7	C24—C23—C28	118.4 (2)
C21—N5—HN5	125.6	C24—C23—C22	106.7 (2)
C30—N6—C37	109.19 (19)	C28—C23—C22	134.8 (2)
C30—N6—HN6	125.5	N5—C24—C25	129.7 (2)
C37—N6—HN6	125.3	N5—C24—C23	107.7 (2)
Ni1—O1—H1A	108.1	C25—C24—C23	122.5 (2)
Ni1—O1—H1B	116.3	C26—C25—C24	117.1 (2)
H1A—O1—H1B	97.8	C26—C25—H25	121.4
H2A—O2—H2B	106.3	C24—C25—H25	121.4
C29—O3—Ni1	128.90 (17)	C25—C26—C27	121.5 (2)
H7B—O7—H7A	118.6	C25—C26—H26	119.3
N1—C1—C2	122.9 (3)	C27—C26—H26	119.3
N1—C1—H1	118.5	C28—C27—C26	121.4 (2)
C2—C1—H1	118.5	C28—C27—H27	119.3
C3—C2—C1	118.1 (3)	C26—C27—H27	119.3
C3—C2—H2	121.0	C27—C28—C23	119.0 (3)
C1—C2—H2	121.0	C27—C28—H28	120.5
C2—C3—C4	120.2 (2)	C23—C28—H28	120.5
C2—C3—H3	119.9	O4—C29—O3	126.4 (2)
C4—C3—H3	119.9	O4—C29—C21	118.1 (2)
C3—C4—C5	118.6 (3)	O3—C29—C21	115.5 (2)
C3—C4—H4	120.7	C31—C30—N6	108.7 (2)
C5—C4—H4	120.7	C31—C30—C38	129.2 (2)
N1—C5—C4	121.7 (2)	N6—C30—C38	121.8 (2)
N1—C5—C6	115.3 (2)	C30—C31—C32	107.8 (2)
C4—C5—C6	123.1 (2)	C30—C31—H31	126.1
N2—C6—C7	121.1 (2)	C32—C31—H31	126.1
N2—C6—C5	115.1 (2)	C33—C32—C37	118.5 (2)
C7—C6—C5	123.8 (2)	C33—C32—C31	134.9 (2)
C8—C7—C6	119.4 (2)	C37—C32—C31	106.6 (2)
C8—C7—H7	120.3	C34—C33—C32	119.0 (2)
C6—C7—H7	120.3	C34—C33—H33	120.5
C9—C8—C7	119.1 (3)	C32—C33—H33	120.5
C9—C8—H8	120.5	C33—C34—C35	121.3 (2)
C7—C8—H8	120.5	C33—C34—H34	119.3
C10—C9—C8	118.6 (3)	C35—C34—H34	119.3
C10—C9—H9	120.7	C36—C35—C34	121.6 (2)
C8—C9—H9	120.7	C36—C35—H35	119.2
N2—C10—C9	122.7 (2)	C34—C35—H35	119.2
N2—C10—H10	118.6	C35—C36—C37	117.1 (2)
C9—C10—H10	118.6	C35—C36—H36	121.4
N3—C11—C12	122.8 (3)	C37—C36—H36	121.4
N3—C11—H11	118.6	N6—C37—C36	129.9 (2)
C12—C11—H11	118.6	N6—C37—C32	107.7 (2)
C11—C12—C13	118.8 (3)	C36—C37—C32	122.4 (2)
C11—C12—H12	120.6	O6—C38—O5	124.6 (2)
C13—C12—H12	120.6	O6—C38—C30	118.0 (2)
C12—C13—C14	119.6 (2)	O5—C38—C30	117.4 (2)
C12—C13—H13	120.2		
			
N4—Ni1—N1—C1	6.3 (2)	N3—C11—C12—C13	0.4 (4)
O1—Ni1—N1—C1	175.6 (4)	C11—C12—C13—C14	0.0 (4)
O3—Ni1—N1—C1	−87.2 (2)	C12—C13—C14—C15	−1.5 (4)
N2—Ni1—N1—C1	−179.5 (2)	C11—N3—C15—C14	−2.5 (3)
N3—Ni1—N1—C1	84.9 (2)	Ni1—N3—C15—C14	176.99 (17)
N4—Ni1—N1—C5	−171.13 (16)	C11—N3—C15—C16	177.54 (19)
O1—Ni1—N1—C5	−1.8 (5)	Ni1—N3—C15—C16	−3.0 (2)
O3—Ni1—N1—C5	95.41 (16)	C13—C14—C15—N3	2.8 (3)
N2—Ni1—N1—C5	3.11 (15)	C13—C14—C15—C16	−177.2 (2)
N3—Ni1—N1—C5	−92.48 (16)	C20—N4—C16—C17	−2.4 (3)
N4—Ni1—N2—C10	−139.6 (4)	Ni1—N4—C16—C17	173.42 (16)
O1—Ni1—N2—C10	−2.1 (2)	C20—N4—C16—C15	179.22 (19)
O3—Ni1—N2—C10	88.3 (2)	Ni1—N4—C16—C15	−5.0 (2)
N1—Ni1—N2—C10	178.5 (2)	N3—C15—C16—N4	5.3 (3)
N3—Ni1—N2—C10	−91.8 (2)	C14—C15—C16—N4	−174.7 (2)
N4—Ni1—N2—C6	37.9 (5)	N3—C15—C16—C17	−173.1 (2)
O1—Ni1—N2—C6	175.46 (16)	C14—C15—C16—C17	6.9 (3)
O3—Ni1—N2—C6	−94.10 (17)	N4—C16—C17—C18	2.3 (3)
N1—Ni1—N2—C6	−3.88 (16)	C15—C16—C17—C18	−179.4 (2)
N3—Ni1—N2—C6	85.80 (17)	C16—C17—C18—C19	−0.2 (4)
N4—Ni1—N3—C11	179.7 (2)	C17—C18—C19—C20	−1.8 (4)
O1—Ni1—N3—C11	−87.7 (2)	C16—N4—C20—C19	0.3 (3)
O3—Ni1—N3—C11	−174.4 (4)	Ni1—N4—C20—C19	−175.07 (18)
N2—Ni1—N3—C11	6.3 (2)	C18—C19—C20—N4	1.8 (4)
N1—Ni1—N3—C11	84.4 (2)	C24—N5—C21—C22	−0.6 (3)
N4—Ni1—N3—C15	0.30 (14)	C24—N5—C21—C29	175.2 (2)
O1—Ni1—N3—C15	92.81 (15)	N5—C21—C22—C23	0.9 (3)
O3—Ni1—N3—C15	6.1 (6)	C29—C21—C22—C23	−174.3 (2)
N2—Ni1—N3—C15	−173.19 (15)	C21—C22—C23—C24	−0.9 (3)
N1—Ni1—N3—C15	−95.04 (16)	C21—C22—C23—C28	176.7 (3)
O1—Ni1—N4—C20	89.34 (19)	C21—N5—C24—C25	−179.7 (3)
O3—Ni1—N4—C20	−1.07 (19)	C21—N5—C24—C23	0.0 (3)
N2—Ni1—N4—C20	−133.1 (4)	C28—C23—C24—N5	−177.5 (2)
N1—Ni1—N4—C20	−92.11 (19)	C22—C23—C24—N5	0.6 (3)
N3—Ni1—N4—C20	178.1 (2)	C28—C23—C24—C25	2.1 (4)
O1—Ni1—N4—C16	−86.09 (15)	C22—C23—C24—C25	−179.7 (2)
O3—Ni1—N4—C16	−176.50 (15)	N5—C24—C25—C26	179.0 (3)
N2—Ni1—N4—C16	51.5 (5)	C23—C24—C25—C26	−0.6 (4)
N1—Ni1—N4—C16	92.46 (15)	C24—C25—C26—C27	−0.8 (4)
N3—Ni1—N4—C16	2.69 (14)	C25—C26—C27—C28	0.7 (4)
N4—Ni1—O3—C29	109.4 (2)	C26—C27—C28—C23	0.9 (4)
O1—Ni1—O3—C29	17.0 (2)	C24—C23—C28—C27	−2.2 (4)
N2—Ni1—O3—C29	−77.1 (2)	C22—C23—C28—C27	−179.7 (3)
N1—Ni1—O3—C29	−155.2 (2)	Ni1—O3—C29—O4	−0.3 (4)
N3—Ni1—O3—C29	103.6 (5)	Ni1—O3—C29—C21	179.74 (15)
C5—N1—C1—C2	−1.3 (4)	C22—C21—C29—O4	169.1 (3)
Ni1—N1—C1—C2	−178.66 (19)	N5—C21—C29—O4	−5.6 (4)
N1—C1—C2—C3	0.5 (4)	C22—C21—C29—O3	−10.9 (4)
C1—C2—C3—C4	0.3 (4)	N5—C21—C29—O3	174.3 (2)
C2—C3—C4—C5	−0.2 (4)	C37—N6—C30—C31	0.1 (3)
C1—N1—C5—C4	1.4 (3)	C37—N6—C30—C38	175.52 (19)
Ni1—N1—C5—C4	179.07 (18)	N6—C30—C31—C32	0.5 (3)
C1—N1—C5—C6	−179.6 (2)	C38—C30—C31—C32	−174.5 (2)
Ni1—N1—C5—C6	−2.0 (2)	C30—C31—C32—C33	179.7 (3)
C3—C4—C5—N1	−0.7 (4)	C30—C31—C32—C37	−0.8 (3)
C3—C4—C5—C6	−179.6 (2)	C37—C32—C33—C34	−2.7 (3)
C10—N2—C6—C7	1.4 (3)	C31—C32—C33—C34	176.8 (3)
Ni1—N2—C6—C7	−176.33 (18)	C32—C33—C34—C35	2.1 (4)
C10—N2—C6—C5	−178.3 (2)	C33—C34—C35—C36	−0.7 (4)
Ni1—N2—C6—C5	4.0 (2)	C34—C35—C36—C37	0.0 (4)
N1—C5—C6—N2	−1.3 (3)	C30—N6—C37—C36	177.7 (2)
C4—C5—C6—N2	177.6 (2)	C30—N6—C37—C32	−0.6 (2)
N1—C5—C6—C7	179.0 (2)	C35—C36—C37—N6	−178.7 (2)
C4—C5—C6—C7	−2.0 (4)	C35—C36—C37—C32	−0.7 (3)
N2—C6—C7—C8	−1.8 (4)	C33—C32—C37—N6	−179.6 (2)
C5—C6—C7—C8	177.9 (2)	C31—C32—C37—N6	0.9 (2)
C6—C7—C8—C9	0.8 (4)	C33—C32—C37—C36	2.0 (3)
C7—C8—C9—C10	0.4 (4)	C31—C32—C37—C36	−177.6 (2)
C6—N2—C10—C9	−0.1 (4)	C31—C30—C38—O6	162.5 (2)
Ni1—N2—C10—C9	177.42 (19)	N6—C30—C38—O6	−11.8 (3)
C8—C9—C10—N2	−0.8 (4)	C31—C30—C38—O5	−16.1 (4)
C15—N3—C11—C12	0.9 (4)	N6—C30—C38—O5	169.5 (2)
Ni1—N3—C11—C12	−178.53 (19)		

### Hydrogen-bond geometry (Å, °)

**Table:**

*D*—H···*A*	*D*—H	H···*A*	*D*···*A*	*D*—H···*A*
O1—H1A···O4	0.82	1.89	2.647 (3)	154.0
O2—H2A···O5	0.82	1.91	2.732 (4)	178.3
O2—H2B···O4	0.82	2.10	2.885 (4)	161.7
O7—H7A···O5	0.81	2.42	2.994 (4)	129.0
N6—HN6···O6^i^	0.86	1.99	2.814 (3)	159.0
O1—H1B···O6^ii^	0.82	1.93	2.750 (3)	174.2
O1—H1B···O5^ii^	0.82	2.60	3.166 (3)	127.6
C4—H4···O2^iii^	0.93	2.54	3.400 (5)	155
C14—H14···O7^iv^	0.93	2.41	3.320 (4)	167

Symmetry codes: (i) −*x*+1, −*y*, −*z*+2; (ii) −*x*, −*y*, −*z*+1; (iii) −*x*+1, −*y*+1, −*z*+1; (iv) *x*−1, *y*, *z*−1.
